# Higher metabolic variability increases the risk of depressive disorder in type 2 diabetes mellitus: a longitudinal nationwide cohort study

**DOI:** 10.3389/fpsyt.2023.1217104

**Published:** 2023-07-24

**Authors:** Ji Hyun An, Kyung-do Han, Hong Jin Jeon

**Affiliations:** ^1^Department of Psychiatry, Depression Center, Samsung Medical Center, Sungkyunkwan University School of Medicine, Seoul, Republic of Korea; ^2^Department of Statistics and Actuarial Science, Soongsil University, Seoul, Republic of Korea; ^3^Department of Health Sciences and Technology, Department of Medical Device Management and Research, Department of Clinical Research Design and Evaluation, Samsung Advanced Institute for Health Sciences and Technology, Sungkyunkwan University, Seoul, Republic of Korea

**Keywords:** type 2 diabetes mellitus, metabolic variability, depressive disorder, nationwide, longitudinal cohort

## Abstract

**Background and objectives:**

While variabilities in metabolic parameters (METv) have been linked to adverse health outcomes in type 2 DM, their association with depression is yet to be studied. This research aimed to investigate the association between METv and depressive disorder in patients with type 2 DM.

**Methods:**

The study involved a nationwide cohort of 1,119,631 type 2 DM patients who had undergone three or more serial health examinations between 2005 and 2012. At each visit, body mass index (BMI), fasting glucose (FG), systolic blood pressure (BP), and total cholesterol (TC) were measured and stratified into quartiles, with Q4 being the highest and Q1 the lowest. The risk of depressive disorder was evaluated using Cox proportional hazard regression models, which accounted for METs in the indexes, after adjusting for sex, income status, lifestyle habits, medical comorbidities, DM severity, and baseline levels of BMI, FG, BP, and TC.

**Results:**

During a mean follow-up period of 6.00 ± 2.42 years, 239,477 (21.4%) cases of type 2 DM patients developed depressive disorder. The risk of developing depressive disorder was gradually increased as the number of METv increased (HR 1.18; 95% CI 1.13, 1.23 for the group with the highest METv in all parameters compared to those with the lowest METv in all parameters). In the subgroup analysis, the risk of developing depressive disorder was 43% higher in men (HR 1.43; 95% CI 1.34, 1.51), and 31% higher in those younger than 65 years of age (HR 1.31; 95% CI 1.23, 1.39) in the group with the highest number of METv compared to the group with the lowest number of METv.

**Conclusion:**

In type 2 DM, higher METv was an independent risk factor for depressive disorder. This risk is notably elevated in men and individuals under the age of 65 years.

## Introduction

Type 2 diabetes mellitus (DM) affects a substantial portion of the global population, with an estimated prevalence of 6.4% and projected growth to impact about 300 million individuals by 2025 (1, 2). In addition to the cardiovascular complications traditionally associated with the disease, individuals with DM are also at an elevated risk of developing psychiatric conditions, particularly depression (3). This underscores the need for comprehensive healthcare approaches to address both the physical and mental health needs of patients with DM.

Depression is the most prevalent comorbid psychiatric condition in individuals with type 2 DM, with a prevalence that is approximately twice that of the general population (4). The burden of DM management and diagnosis can engender the onset of depressive symptoms, exacerbating the risk for complications related to hyperglycemia, diabetic complications, poor adherence to DM management, unhealthy lifestyles, and increased mortality rates (5–8). Therefore, early identification and management of depression risk factors hold considerable clinical significance in type 2 DM (9).

Individuals with DM frequently exhibit a range of metabolic syndromes, including obesity, dyslipidemia, hypertension, and insulin resistance, which result from disruptions in glucose homeostasis (10–12). Previous studies have highlighted the importance of metabolic risk management in mitigating adverse health outcomes, including depression (13, 14).

Furthermore, recent studies have suggested that not only the static magnitude of metabolic parameters but also their variabilities over time may increase the risk of adverse health outcomes. High variability in metabolic parameters (METv), such as body weight, blood glucose, cholesterol, or blood pressure, has been associated with cardiovascular disease and mortality in both the general population (15–17) and individuals with type 2 DM (18–20). Such METv tend to exist consistently by individual and has emerged as new disease-modifiable indicators in chronic metabolic diseases such as DM.

A few studies have evaluated the association between depression and METv, including body mass index, lipid, or blood glucose levels. A recent study demonstrated that several METv increased the risk of depression in the general population (21), and BMI variability was associated with depression in individuals with type 2 DM (22). However, the association between depression and a composite of such METv in individuals with DM has not been studied.

In this large nationwide population-based study, we investigated the association between METv and incident depressive disorder in individuals with type 2 DM. We hypothesized that higher METv would increase the risk of depressive disorder.

## Methods

### Data source

This retrospective, longitudinal cohort study utilized data from the Korean National Health Insurance System (NHIS), a comprehensive single insurer operated by the government that includes health information for about 97% of the Korean population (≥51 million). NHIS enrollees are recommended to undergo annual or biennial general health examinations that include standardized assessments of sociodemographic information (age, sex, and level of income), lifestyle habits (alcohol consumption, smoking, and regular exercise), anthropometric measurements, and laboratory tests. The quality of the health examination process is closely monitored by the NHIS, and the health examination data are linked to a nationwide medical claim database that includes clinical diagnoses, medical procedures, prescriptions, and healthcare service utilization as described elsewhere (23, 24). Since the insured essential health services are all registered to the NHIS system, nationwide-scale epidemiological studies dealing with medical outcomes have been vigorously generated using the NHIS database.

### Study population

We identified 2,746,079 individuals with type 2 DM who underwent general health examinations between 1 January 2009 and 12 December 2012 (index year) and had undergone serial health examinations at least twice in the previous 5 years (between 1 January 2005 and 12 December 2008) to measure intra-metabolic variability. Exclusions were made for individuals younger than 20 years old (*n* = 390), missing data (*n* = 78,479), and those who developed depressive disorder within a 1-year lag period (*n* = 230,240), resulting in a final sample size of 1,119,631. The study was approved by the institutional review board of Samsung Medical Center, and informed consent was waived due to the use of de-identified and anonymous information.

### Ascertainment of type 2 DM

Individuals with type 2 DM were identified based on the presence of ≥1 annual claim under *International Classification of Diseases, Tenth Revision (ICD-10)* codes of E11 to E14 and ≥1 annual claim for antidiabetic medication prescriptions, or based on a fasting glucose level of ≥126 mg/dL according to the operational definition of DM provided by the Korean Diabetes Association (25).

### Definition of covariates and measurement

Sociodemographic information, including age, sex, and level of income (presented as quartile), as well as lifestyle habits, such as heavy drinking (defined as ≥30 g of alcohol per day), smoking (defined as current, past, and never), and regular exercise (defined as moderate intensity physical activity more than five times per week or vigorous-intensity physical activity more than three times per week), were obtained via self-questionnaire. Anthropometric measurements, including height, weight, and waist circumference, were obtained using standardized units and protocols. Body mass index (BMI) was calculated as the weight (kg) divided by the square of the height (m^2^). Blood pressure (BP) was measured as both systolic and diastolic pressure by an expert clinician after a 5-min seated rest. Blood sampling for serum fasting glucose and lipid levels and urine analysis were performed after an overnight fast.

We also collected medical covariates, including hypertension (defined as ≥1 annual claim under ICD-10 codes of I10–13 or I15 with a history of antihypertensive medication use, or with systolic/diastolic BP ≥ 140/90 mmHg), dyslipidemia (defined as ≥1 annual claim under ICD-10 codes of E78 with a history of lipid-lowering medication use or with a total cholesterol level ≥240 mg/dL), and chronic kidney disease [defined as estimated glomerular filtration rate (eGFR) of <60 ml/min/1.73 m^2^].

### Definition of metabolic parameters

METv was defined as the intra-individual variability of body mass index (BMI), systolic blood pressure (BP), fasting glucose (FG), and total cholesterol (TC) values measured at each visit of the health examination, as these parameters have been widely studied in relation to metabolic variabilities and health outcomes (15, 21, 26–28). METv was calculated using the variability independent of the mean (VIM) method, which involves calculating the VIM as 100 × SD/mean^β^, where β is the regression coefficient based on the natural logarithm of SD on the natural logarithm of the mean.

Given the absence of a specific cutoff point, we chose to classify the data into quartiles for the Variability Index Measurement (VIM) analysis, which is consistent with its utilization in previous studies (15, 21, 22). Individuals with type 2 DM were allocated into quartiles of METv (Q1 as the lowest, Q4 as the highest). “High” variability was defined as Q4 variability, while “low” variability was defined as the lower Q1–Q3 variability level of each metabolic parameter. A score of 0 indicates no high-variability parameter, while scores of 1–4 indicate the number of high-variability parameters out of the total four parameters. For instance, a score of 3 indicates high variability in three of the four parameters.

To assess the cumulative impact of METv, we assigned 0 points to Q1, 1 point to Q2, 2 points to Q3, and 3 points to Q4 for each of the metabolic parameters and summed the score. This generated a total METv score ranging from 0 to 12 points for each individual, with those scoring 0 points belonging to the Q1 group and those scoring 12 points belonging to the Q4 group for all four metabolic parameters.

### Study outcomes and follow-up

The study endpoint was the diagnosis of newly developed depressive disorder, defined by the *International Classification of Diseases, Tenth Revision (ICD-10)* codes of F32.0–F33.9. To minimize confounding and reverse causality by pre-existing depression, we set a 1-year lag period following the index year. The study cohort was followed until the date of depression diagnosis or death or until the end of the study (31 December 2018).

### Statistical analysis

Baseline characteristics were presented as mean ± standard deviation (SD) or as number (%). For the analysis of continuous variables, we performed ANOVA, while for the analysis of categorical variables, we conducted the chi-square test with the effect size calculated as the absolute standardized mean difference (ASD). Study cohorts were categorized into five groups according to the number of high METv, ranging from 0 to 4. The incidence rate of the primary outcome was calculated by dividing the number of cases by the total duration of follow-up, measured in person-years. The cumulative incidence of the primary outcome for each group was depicted in Kaplan-Meier estimates, due to the large sample size and a balanced distribution of baseline variables. Differences between groups were compared using the log-rank test.

The risk of newly diagnosed depressive disorder was assessed using a multivariable-adjusted Cox proportional hazards model, with results presented as hazard ratios (HR) and 95% confidence intervals (CI). We have assessed the proportional hazard assumption using Schoenfeld's residuals and the Log-Log plot, which confirmed that the assumption is satisfied. No significant deviation from proportionality over time was observed.

Model 1 was adjusted for age and sex. Model 2 was adjusted further for alcohol drinking, smoking, regular exercise, income status, and variables related to the severity of diabetes, including insulin use, the number of oral hypoglycemic agents (more than three medications), and the duration of DM (more than 5 years). Model 3 was finally adjusted further for baseline levels of BMI, BP, FBG, and TC.

Subgroup analyses were performed to explore the potential effect of modifiable confounders, including age, sex, baseline obesity (defined as BMI ≥ 25 kg/m^2^), existing medical comorbidities, and DM severity. Statistical analyses were conducted using the SAS version 9.4 (SAS Institute Inc.), with a significance level set at *p* < 0.05.

## Results

### Baseline characteristics

The study population consisted of 1,119,631 individuals with type 2 DM [762,538 (68.1%) men and 357,093 (31.9%) women]. Baseline characteristics of the study cohort were stratified by the number of high METv and are presented in Table 1. Individuals with high METv tended to be older and female, have lower income, a higher prevalence of hypertension, dyslipidemia, chronic kidney disease, and more advanced DM with higher fasting glucose levels and an increased number of antidiabetic medications or insulin use. In contrast, they exhibited lower mean values of weight, waist circumference, and body mass index (BMI), as well as lower levels of blood pressure and total cholesterol. Individuals with high METv also demonstrated a lower propensity for smoking, drinking, and regular exercise.

**Table 1:** Baseline characteristics of patients with type 2 DM according to the number of high metabolic variabilities.

**Variables mean (±SD) or *n* (%)**	**Metabolic parameters**						**Maximum ASD**
	**0**	**1**	**2**	**3**	**4**	***p***	
	***N** =* **380,297**	***N** =* **438,869**	***N** =* **228,847**	***N** =* **63,508**	***N** =* **8,110**		
Age, years	55.83 ± 11.51	56.45 ± 12.26	57.24 ± 12.84	58.12 ± 13.13	59.3 ± 13.06	<0.0001	0.2819
Sex						<0.0001	
Male	277,533 (72.98)	297,307 (67.74)	144,858 (63.3)	38,102 (60)	4,738 (58.42)		0.3104
Female	102,764 (27.02)	141,562 (32.26)	83,989 (36.7)	25,406 (40)	3,372 (41.58)		0.3104
Income, lowest quartile	67,484 (17.75)	85,696 (19.53)	48,182 (21.05)	13,851 (21.81)	1,803 (22.23)	<0.0001	0.1122
Smoking						<0.0001	
Non	183,991 (48.38)	225,249 (51.32)	123,882 (54.13)	35,591 (56.04)	4,687 (57.79)		0.1894
Ex	91,106 (23.96)	92,468 (21.07)	43,836 (19.16)	11,777 (18.54)	1,439 (17.74)		0.1536
Current	105,200 (27.66)	121,152 (27.61)	61,129 (26.71)	16,140 (25.41)	1,984 (24.46)		0.0729
Drinking						<0.0001	
Non	176,996 (46.54)	227,073 (51.74)	130,082 (56.84)	38,832 (61.15)	5,338 (65.82)		0.3961
Mild	160,503 (42.2)	167,331 (38.13)	77,664 (33.94)	19,282 (30.36)	2,162 (26.66)		0.3315
Heavy	42,798 (11.25)	44,465 (10.13)	21,101 (9.22)	5,394 (8.49)	610 (7.52)		0.1282
Regular exercise	91,546 (24.07)	98,804 (22.51)	47,987 (20.97)	12,603 (19.84)	1,524 (18.79)	<0.0001	0.1289
BMI, kg/m^2^	25.19 ± 3.04	25.01 ± 3.28	24.88 ± 3.51	24.69 ± 3.68	24.42 ± 3.83	<0.0001	0.2227
WC, cm	85.82 ± 7.98	85.38 ± 8.42	85.09 ± 8.81	84.76 ± 9.11	84.49 ± 9.28	<0.0001	0.1537
Systolic BP, mmHg	129.56 ± 13.47	128.65 ± 15.21	127.86 ± 16.68	127.03 ± 18.27	126.35 ± 19.86	<0.0001	0.1892
Diastolic BP, mmHg	79.85 ± 9.44	79.04 ± 9.97	78.43 ± 10.5	77.83 ± 11.16	77.2 ± 11.76	<0.0001	0.2485
Fasting glucose, mg/dL	139.39 ± 33.23	143.16 ± 42.29	146.27 ± 51.57	148.98 ± 60.7	150.9 ± 69.2	<0.0001	0.212
Total cholesterol, mg/dL	200.84 ± 36.43	195.35 ± 41.02	191.92 ± 45.6	188.98 ± 50.48	185.72 ± 54.51	<0.0001	0.3261
HDL-C, mg/dL	51.58 ± 20.89	51.9 ± 22.6	51.99 ± 24.14	52 ± 25.58	51.84 ± 26.44	<0.0001	0.0176
LDL-C, mg/dL	115.33 ± 36.98	110.06 ± 40.14	106.9 ± 43.06	104.03 ± 46.54	101.24 ± 49.52	<0.0001	0.3224
Triglyceride, mg/dL	149.31 (149.04–149.58)	145.83 (145.58–146.08)	143.38 (143.03–143.73)	141.98 (141.32–142.65)	139.88 (138.04–141.74)	<0.0001	0.111
Hypertension	192,857 (50.71)	237,571 (54.13)	131,429 (57.43)	38,549 (60.7)	5,260 (64.86)	<0.0001	0.2895
Dyslipidemia	120,891 (31.79)	172,843 (39.38)	105,425 (46.07)	33,008 (51.97)	4,597 (56.68)	<0.0001	0.5177
Chronic kidney disease	31,965 (8.41)	42,399 (9.66)	26,631 (11.64)	9,039 (14.23)	1,478 (18.22)	<0.0001	0.2918
DM duration, ≥5 years	98,965 (26.02)	123,739 (28.19)	69,458 (30.35)	21,084 (33.2)	2,964 (36.55)	<0.0001	0.2286
Insulin	17,396 (4.57)	28,548 (6.5)	21,292 (9.3)	8,386 (13.2)	1,542 (19.01)	<0.0001	0.4594
Use of OHA ≥ 3	40,124 (10.55)	56,444 (12.86)	35,507 (15.52)	11,682 (18.39)	1,702 (20.99)	<0.0001	0.2894

BMI, body mass index; WC, weight circumference; BP, blood pressure; HDL-C, high density lipoprotein-cholesterol; LDL-C, low density lipoprotein-cholesterol; OHA, oral antihyperglycemic agent; ASD, absolute standardized mean difference.

### Risk of the depressive disorder according to the variability of each metabolic parameters

During a mean follow-up period of 6.00 ± 2.42 (mean ± SD) years, 239,477 (21.4%) cases of newly diagnosed depressive disorder were observed. Incremental increases in the risk of depressive disorder were observed with increasing quartiles of each METv parameter in individuals with type 2 DM (Table 2). Compared to the lowest quartile (reference), the highest quartile of BMI variability increased the risk of depressive disorder by 12% (HR 1.10; 95% CI 1.10, 1.13), the highest quartile of SBP increased the risk by 6% (HR 1.06; 95% CI 1.04, 1.07), the highest quartile of fasting glucose increased the risk by 3% (HR 1.03; 95% CI 1.02, 1.04), and the highest quartile of total cholesterol increased the risk by 11% (HR 1.11; 95% CI 1.09, 1.12).

**Table 2:** Adjusted HR and 95% CI of depressive disorder by quartiles of variabilities in metabolic parameters in type 2 DM.

	***N***	**Event**	**PY (Duration)**	**IR, per 1,000 PY**	**Adjusted HR (95% CI)**		
					**Model 1**	**Model 2**	**Model 3**
**Body mass index (BMI)**							
Q1	279,907	57,195	1,983,842.8	28.8	1 (Ref.)	1 (Ref.)	1(Ref.)
Q2	279,898	56,036	1,994,767.0	28.1	**1.01 (1.00, 1.02)**	**1.01 (1.00, 1.03)**	**1.01 (1.00, 1.02)**
Q3	279,918	59,767	1,965,340.2	30.4	**1.07 (1.06, 1.08)**	**1.06 (1.05, 1.08)**	**1.06 (1.05, 1.07)**
Q4	279,908	66,479	1,889,114.9	35.2	**1.14 (1.13, 1.16)**	**1.12 (1.11, 1.13)**	**1.12 (1.10, 1.13)**
**Blood pressure (SBP)**							
Q1	281,137	57,774	1,975,077.9	29.3	1 (Ref.)	1 (Ref.)	1 (Ref.)
Q2	278,678	55,093	1,979,652.5	27.8	0.99 (0.98, 1.00)	0.99 (0.98, 1.01)	0.99 (0.98, 1.00)
Q3	280,013	58,923	1,972,161.1	29.9	**1.02 (1.01, 1.03)**	**1.02 (1.01, 1.03)**	**1.02 (1.01, 1.03)**
Q4	279,803	67,687	1,906,173.4	35.5	**1.08 (1.06, 1.09)**	**1.06 (1.05, 1.08)**	**1.06 (1.04, 1.07)**
**Fasting glucose (FG)**							
Q1	279,901	64,944	1,926,403.4	33.7	1 (Ref.)	1 (Ref.)	1 (Ref.)
Q2	279,905	60,057	1,956,491.8	30.7	0.99 (0.98, 1.00)	0.99 (0.98, 1.00)	0.99 (0.98, 1.00)
Q3	279,917	58,097	1,971,178.4	29.5	**1.02 (1.01, 1.03)**	**1.02 (1.01, 1.03)**	**1.02 (1.01, 1.03)**
Q4	279,908	56,379	1,978,991.3	28.5	**1.05 (1.04, 1.06)**	**1.03 (1.02, 1.04)**	**1.03 (1.02, 1.04)**
**Total cholesterol (TC)**							
Q1	279,907	54,543	1,980,871.5	27.5	1 (Ref.)	1 (Ref.)	1 (Ref.)
Q2	279,908	54,650	1,992,430.3	27.4	**1.03 (1.02, 1.04)**	**1.03 (1.02, 1.04)**	**1.03 (1.02, 1.04)**
Q3	279,908	59,963	1,962,731.3	30.6	**1.09 (1.08, 1.10)**	**1.07 (1.06, 1.08)**	**1.07 (1.06, 1.08)**
Q4	279,908	70,321	1,897,031.7	37.1	**1.18 (1.16, 1.19)**	**1.11 (1.09, 1.12)**	**1.11 (1.09, 1.12)**

PY, person-year; IR, incident rate; HR, hazard ratio; CI, confidence interval.

Model 1: adjusted for age and sex.

Model 2: adjusted for Model 1 + smoking status, alcohol drinking, regular exercise, income level, and medical comorbidities.

Model 3: adjusted for Model 2 + baseline level of BMI, SBP, FG, and TC. Values highlighted in bold indicate statistical significance.

### Risk of the depressive disorder according to the number of highest variability metabolic parameters

**Figure 1:**
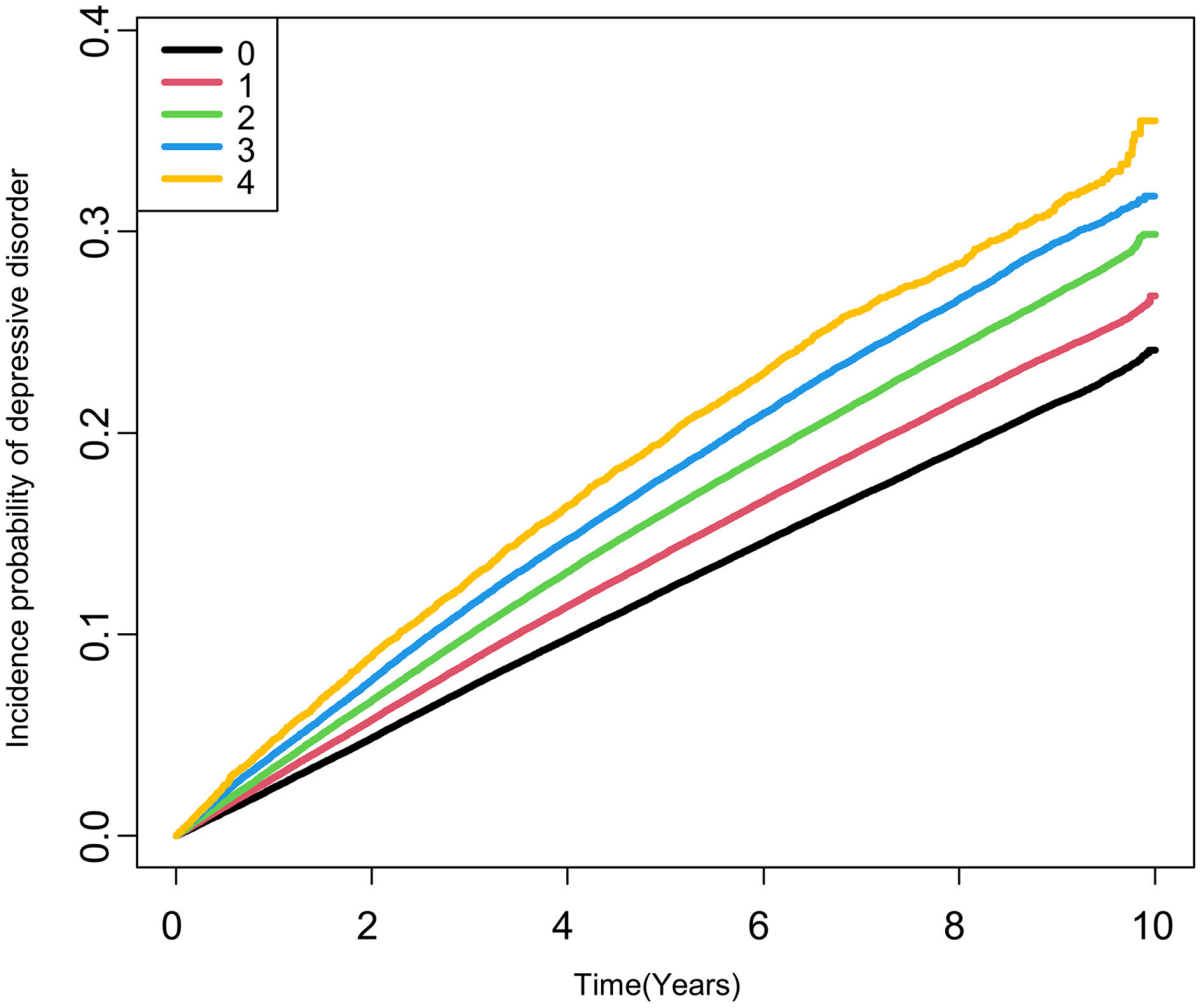
Kaplan-Meier estimates of cumulative incidence of depressive disorder according to the number of high metabolic variability in type 2 DM. High metabolic variabilities were defined as the fourth quartile of variabilities in each metabolic parameter.

In individuals with type 2 DM, the risk of depressive disorder increased as the total number of high METv increased (Figure 1; Table 3). Compared to the group with the lowest number of high METv, the group with the highest number of high METv had a significantly higher risk of depressive disorder (HR 1.18; 95% CI 1.13, 1.23).

**Table 3:** Adjusted HR and 95 % CI of depressive disorder by summed number of high metabolic variability type 2 DM.

**Number of metabolic variabilities**	***N***	**Event**	**PY (Duration)**	**IR, per 1,000 PY**	**Adjusted HR (95% CI)**		
					**Model 1**	**Model 2**	**Model 3**
0	380,297	72,605	2,721,622.6	26.7	1 (Ref.)	1 (Ref.)	1 (Ref.)
1	438,869	93,709	3,076,177.5	30.5	**1.08 (1.07, 1.10)**	**1.06 (1.05, 1.07)**	**1.06 (1.05, 1.07)**
2	228,847	54,546	1,562,461.4	34.9	**1.18 (1.17, 1.19)**	**1.13 (1.11, 1.15)**	**1.12 (1.11, 1.14)**
3	63,508	16,403	421,002.9	39.0	**1.26 (1.24, 1.28)**	**1.17 (1.15, 1.19)**	**1.17 (1.15, 1.19)**
4	8,110	2,214	51,800.8	42.7	**1.32 (1.26, 1.38)**	**1.19 (1.14, 1.24)**	**1.18 (1.13, 1.23)**

PY, person-year; IR, incident rate; HR, hazard ratio; CI, confidence interval.

Model 1: adjusted for age and sex.

Model 2: adjusted for Model 1 + smoking status, alcohol drinking, regular exercise, income level, and medical comorbidities.

Model 3: adjusted for Model 2 + baseline level of BMI, SBP, FG, and TC. Values highlighted in bold indicate statistical significance.

**Figure 2:**
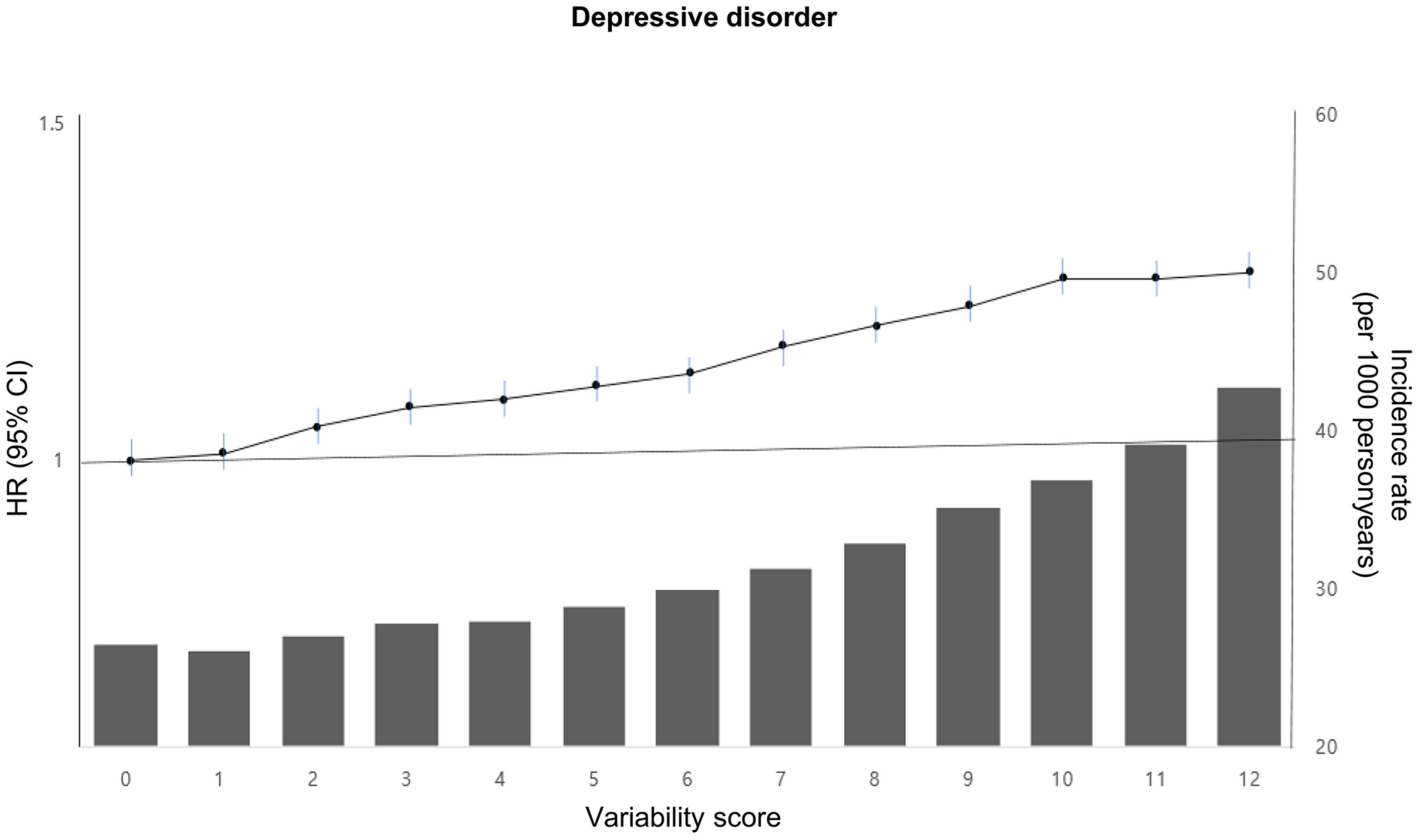
Incidence rate, adjusted HR, and 95% CI of depressive disorder according to the metabolic variability score in type 2 DM. The variability score for each of the four parameters (BMI, SBP, FG, and TC) was calculated by assigning points based on quartiles of variability. The lowest quartile (Q1) received 0 points, the second quartile (Q2) received 1 point, the third quartile (Q3) received 2 points, and the highest quartile (Q4) received 3 points. The total points were then summed for each individual to obtain a variability score ranging from 0 to 12. This score was adjusted for age, sex, alcohol drinking, smoking, regular exercise, income status, medical comorbidities, DM medications, and baseline levels of BMI, SBP, FG, and TC.

The incidence rate and risk of depressive disorder showed a linear increase as the METv score increased (ranging from 0 to 12 points), with a 28% increased risk (HR 1.28; 95% CI 1.19, 1.37) observed in the group with 12 points compared to the group with 0 points (Figure 2; Supplementary Table 1).

### Subgroup analysis

**Figure 3:**
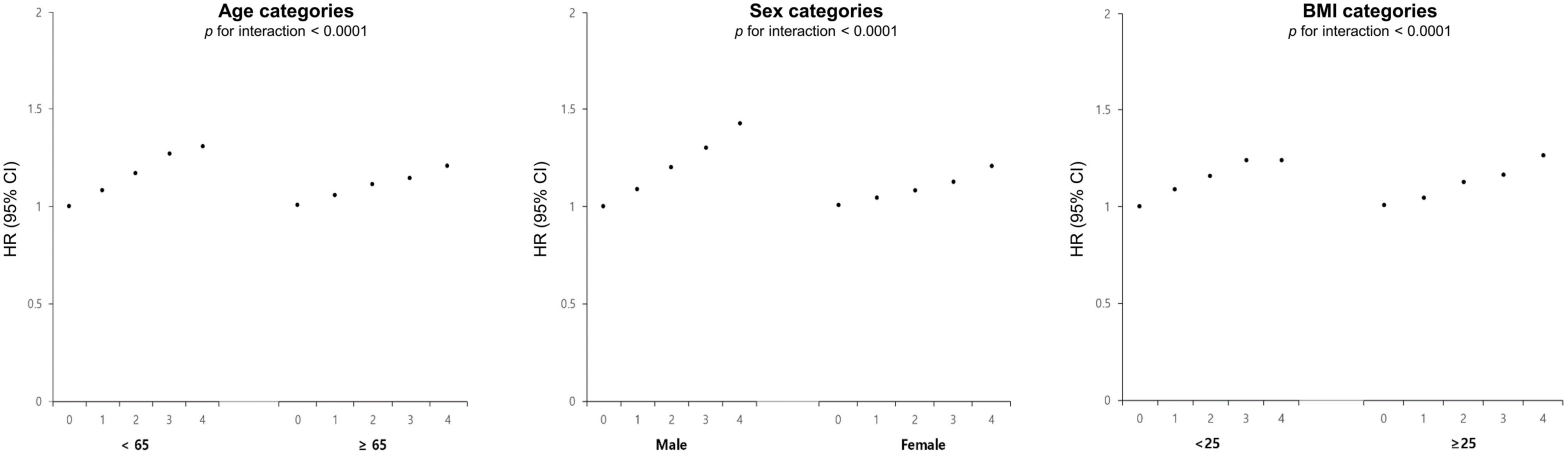
Adjusted HR and 95% CI of depressive disorder stratified by age, sex, and BMI categories according to the number of high metabolic variability in type 2 DM. This score was adjusted for age, sex, alcohol drinking, smoking, regular exercise, income status, medical comorbidities, DM medications, and baseline levels of BMI, SBP, FG, and TC.

Figure 3 depicts the risk of newly diagnosed depressive disorder based on age, sex, and BMI stratification, along with the number of high METv. The analysis showed higher adjusted hazard ratios (HR) of depression in subgroups with age <65 years, male, and obese. Specifically, the male subgroup had a 43% increased risk of depressive disorder (HR 1.43; 95% CI 1.34, 1.51), while the subgroup <65 years had a 31% increased risk (HR 1.31; 95% CI 1.23, 1.39) in the group with the highest number of high METv, compared to the group with the least number of high METv. In addition, the subgroup with the presence of medical comorbidities including chronic kidney disease and a longer duration of DM also showed a greater risk of depressive disorder as the number of metabolic variabilities increased (Supplementary Table 2).

## Discussion

This study demonstrated the association between higher variability in BMI, SBP, FBG, and TC and higher risk for the development of depressive disorder in individuals with type 2 DM. We further found that the number of elevated metabolic variabilities was associated with new-onset depressive disorder in a graded manner. These associations persisted after multivariable adjustment including baseline metabolic parameters and medical comorbidities. Also, the effects of metabolic variability on depressive disorder were more pronounced in both men and those aged under 65 years.

In this study, 22% of individuals with type 2 DM have developed incident depression, which is comparable to previous studies (29). A number of biological processes, as well as the emotional disease burden, have been suggested to explain the high prevalence of depression in DM. Impaired insulin-glucose homeostasis cause activation of the hypothalamic-pituitary-adrenal (HPA) axis, dysfunction in the autonomic nervous system, or a pro-inflammatory state, and consequently alteration in metabolic networks (30–34). It may affect the production and transport of mood-regulatory neurotransmitters, such as brain-derived neurotrophic factor, neural growth, change in cerebral blood flow, and microvascular damage, which are known to be potential etiological mechanisms of depression (35–37). Also, the presence of coexisting metabolic syndrome in DM may hasten the onset of depression (38–40).

This study further highlighted that the composite of METv in type 2 DM was independently associated with incident depression even after adjusting the presence of metabolic syndromes. METv in type 2 DM has already been recognized as a critical concern, associated with an increased risk of medical comorbidities such as cardiovascular events, dementia, and elevated mortality rates (41, 42), but our study advances current knowledge by uncovering a significant association between METv and psychiatric symptoms, particularly depression in type 2 DM patients. While there is one comparable study demonstrating a similar association in non-diabetic populations (21), it is vital to underscore that metabolic variabilities are more prominent within metabolic disorders like diabetes and present a potentially modifiable target.

The development of metabolic variability in patients with DM is a complex and multi-factorial process that may involve an interplay between genetic and environmental factors (43–46). Also, the lifestyle factors, such as an unhealthy diet and physical inactivity and changes in the treatment regimen, including changes in insulin dose or the addition of other medications, can also impact the metabolic status of these individuals. It is imperative to recognize the bidirectional relationship between lifestyle changes, stigmatization of diabetes, emotional burden, and metabolic status, as it can ultimately lead to the development of depression.

The underlying mechanisms linking metabolic variability and depression are not well-understood. A few studies have postulated possible mechanisms of depression in each independent metabolic variability. Increased glucose variability might be related to changes in insulin sensitivity, insulin secretion, and increased oxidative stress on the endothelial function, which results in atherosclerotic change (47, 48). Blood pressure variability is an independent risk of micro-macrovascular complications and can impair cerebral blood flow (49, 50). BMI variabilities involve changes in appetite regulation, energy metabolism, and neuroendocrine function that occur with depression (22, 51). Cholesterol variability cause dysfunction in lipid metabolism, altering cellular membrane function, impairment in the production of stress hormones (45, 52). These variabilities result in alterations in neuronal plasticity, cellular architecture, decreased cellular resilience, or structural abnormalities in the brain (53, 54).

Rather than ascribing the complex etiology of depression to a singular factor, it is more prudent to contemplate a multifactorial approach that incorporates various metabolic variabilities, given their coexistence and the interdependent relationship between multiple metabolic parameters. Notably, changes in one metabolic factor can exert an effect on other interconnected metabolic factors, such as the association between glycemic change and blood pressure fluctuations (55–57). Further research is warranted to ascertain the precise nature of these associations, elucidate whether they are direct or indirect, and discern whether they stem from shared risk factors.

Furthermore, metabolic variability may serve as a clinical marker of underlying frailty or incipient stages of disease. Given the bidirectional association between DM and depression, wherein each condition can impact the other, metabolic variability may potentially represent a prodromal or early phase of depression (10, 58, 59). Additionally, as depression can trigger disruptions in neuroendocrine or immune function, the identification of metabolic variability could hold considerable value as an early predictor of depressive symptoms (60, 61).

The subgroup analysis revealed that high metabolic variability confers a greater risk of depression in men with type 2 DM, increasing it by approximately 40%. The elevated risk of depression in men with high metabolic variability may be due to several mechanisms. While depression is typically more prevalent in women, the negative health impact of metabolic syndrome is often more pronounced in women than in men (62). However, given the inherent metabolic vulnerability in DM (63), it appears that resilience to metabolic variability may be more reduced in men. Differences in hormonal factors, including visceral adipocyte activity, adrenergic stimulation, and sex hormones, may contribute to a higher risk of depression in men with DM (64, 65). Additionally, sex-specific differences in the pathophysiology of DM, such as those related to glucose and lipid metabolism, may further influence the association between depression and DM (66). However, women with type 2 DM may have a higher prevalence of comorbidities, such as cardiovascular disease and metabolic syndrome, which can exacerbate the impact of metabolic variability on the risk of depression (67). These findings underscore the need for gender-specific research in the identification and management of metabolic factors that may influence depression risk in DM.

Our findings also suggest that the impact of metabolic variability on depression is particularly notable in individuals under the age of 65 years. While the risk of metabolic disease, including type 2 DM, tends to increase with age due to declining metabolic homeostasis regulation (68, 69), recent research suggests that younger individuals may experience a higher level of metabolic variability (70, 71). This variability may arise from a range of lifestyle factors, including poor dietary habits, high calorie and fat intake, physical inactivity, and irregular sleep patterns, as well as genetic predisposition such as insulin resistance, which is particularly relevant to metabolic variability in younger age groups (72). These results highlight the complex interplay of genetic and environmental factors in shaping metabolic variability in younger individuals, emphasizing the critical need for early detection of metabolic disturbances and lifestyle interventions in this population.

### Strengths and limitations

This is the first study to investigate the association between metabolic variability and the development of depression in type 2 DM. Moreover, the study entailed a substantial sample size of DM patients at a national level, with systematic and repeated assessments of metabolic parameters. In addition, the investigation examined not only a single factor but also a combination of multiple metabolic variabilities, which better represent real-life situations.

There are several limitations to this study. First, the diagnosis of depression was based on claim data, which may have underestimated the true prevalence of depression compared to clinically diagnosed cases. Second, although we excluded prior depression and applied a lag period, retrospective study designs like ours may be limited in detecting reverse causality between depression and DM. Future research should consider extending lag periods and utilizing prospective designs for a more robust causality analysis. Furthermore, the prodromal phase of depression may not have been fully captured in our study. Third, our investigation focused exclusively on the amplitude of metabolic variabilities and did not examine their directional impact, such as increased, decreased, or sustained changes. As such, future research is warranted to determine whether the direction of metabolic changes may influence the development of depression. Fourth, unmeasured confounders may exist, which could impact the development of depression. Finally, our study population consisted of individuals covered by NHIS and therefore did not include individuals who are not covered by this healthcare system.

## Conclusion

In type 2 DM, the variability of metabolic parameters was an independent risk factor for depressive disorder. This risk is notably elevated in men and individuals under the age of 65 years. Thorough comprehension of the underlying mechanisms responsible for metabolic variability is pivotal for the successful management of depression in patients with type 2 DM.

## Data availability statement

The raw data supporting the conclusions of this article will be made available by the authors, without undue reservation.

## Ethics statement

The studies involving human participants were reviewed and approved by the Institutional Review Board of Samsung Medical Center. Written informed consent for participation was not required for this study in accordance with the national legislation and the institutional requirements.

## Author contributions

JHA participated in the study design, conception, wrote the first manuscript drafting, and revised new drafts from KDH and HJJ. KDH conceptualized the study and participated in the directed acquisition of the data and data analysis. HJJ participated in the whole study design, conception, and manuscript drafting. All authors read and approved the final manuscript.
