# Supplementary material for: Higher metabolic variability increases the risk of depressive disorder in type 2 diabetes mellitus: a longitudinal nationwide cohort study
Source: Front Psychiatry. 2023 Jul 24;14:1217104. doi: 10.3389/fpsyt.2023.1217104 (PMC10405173; doi:10.3389/fpsyt.2023.1217104)
Supplement: Supplementary file 1 [file Table_1.docx]

**Supplementary Materials**

**Supplementary Table 1. Incidence rate and adjusted HR of depressive disorder according to the metabolic variability score**

| **Score of metabolic variability** | **N** | **Event** | **PY**  **(Duration)** | **IR,  per 1000 PY** | **Adjusted HR (95% C.I)** | | |  |
| --- | --- | --- | --- | --- | --- | --- | --- | --- |
|  |  |  |  |  | **Model 1** | **Model 2** | **Model 3** | |
| 0 | 6813 | 1279 | 48,357.8 | 26.4487 | 1 (Ref.) | 1 (Ref.) | 1 (Ref.) | |
| 1 | 23078 | 4284 | 164,001.1 | 26.1218 | 1.01 (0.95, 1.08) | 1.01 (0.95, 1.07) | 1.01 (0.95, 1.07) | |
| 2 | 51235 | 9861 | 365,696.1 | 26.965 | **1.06 (1.00, 1.12)** | **1.05 (1.00, 1.12)** | 1.05 (0.99, 1.12) | |
| 3 | 92267 | 18217 | 655,875.9 | 27.7751 | **1.10 (1.04, 1.16)** | **1.09 (1.03, 1.15)** | **1.08 (1.02, 1.15)** | |
| 4 | 133679 | 26490 | 948,544.0 | 27.927 | **1.11 (1.05, 1.17)** | **1.09 (1.03, 1.16)** | **1.09 (1.03, 1.15)** | |
| 5 | 164721 | 33521 | 1,164,981.7 | 28.7738 | **1.14 (1.08, 1.20)** | **1.12 (1.06, 1.18)** | **1.11 (1.05, 1.17)** | |
| 6 | 179307 | 37659 | 1,261,142.3 | 29.861 | **1.17 (1.11, 1.24)** | **1.15 (1.09, 1.21)** | **1.13 (1.07, 1.20)** | |
| 7 | 163313 | 35559 | 1,140,920.4 | 31.1669 | **1.22 (1.15, 1.29)** | **1.18 (1.12, 1.25)** | **1.17 (1.10, 1.23)** | |
| 8 | 132161 | 29996 | 915,091.6 | 32.7792 | **1.28 (1.19, 1.33)** | **1.22 (1.15, 1.30)** | **1.20 (1.13, 1.26)** | |
| 9 | 90967 | 21782 | 620,762.2 | 35.0891 | **1.31 (1.24, 1.39)** | **1.26 (1.19, 1.34)** | **1.23 (1.16, 1.30)** | |
| 10 | 50312 | 12494 | 339,307.4 | 36.8221 | **1.36 (1.29, 1.44)** | **1.31 (1.24, 1.39)** | **1.27 (1.20, 1.34)** | |
| 11 | 23668 | 6121 | 156,583.7 | 39.0909 | **1.40 (1.31, 1.48)** | **1.33 (1.25, 1.41)** | **1.27 (1.20, 1.35)** | |
| 12 | 8110 | 2214 | 51,800.8 | 42.7407 | **1.44 (1.34, 1.54)** | **1.36 (1.27, 1.45)** | **1.28 (1.19, 1.37)** | |

PY, person-year; IR, Incident rate

Model 1: adjusted for age and sex.

Model 2: adjusted for Model 1 + smoking status, alcohol drinking, regular exercise, income level and medical comorbidities.

Model 3: adjusted for Model 2 + baseline level of BMI, SBP, FG and TC

**Supplementary Table 2. Subgroup analysis of incidence rate and adjusted HR of depressive disorder in type 2 DM.**

|  |  | **Number of metabolic VIM Q4** | **N** | **Event** | **Duration** | **IR, per 1000 PY** | **HR (95% C.I)** | ***p* for interaction** |
| --- | --- | --- | --- | --- | --- | --- | --- | --- |
| **Age** | **<65 years** | 0 | 287758 | 43293 | 2,126,191.8 | 20.4 | 1 (Ref.) | **<.0001** |
|  |  | 1 | 317806 | 52796 | 2,323,120.5 | 22.7 | **1.08 (1.07, 1.09)** |  |
|  |  | 2 | 157742 | 28928 | 1,136,518.3 | 25.5 | **1.17 (1.16, 1.19)** |  |
|  |  | 3 | 41726 | 8370 | 295,356.4 | 28.3 | **1.27 (1.24, 1.30)** |  |
|  |  | 4 | 5032 | 1050 | 34,854.3 | 30.1 | **1.31 (1.23, 1.39)** |  |
|  | **≥65 years** | 0 | 92539 | 29312 | 595,430.8 | 49.2 | 1 (Ref.) |  |
|  |  | 1 | 121063 | 40913 | 753,057.0 | 54.3 | **1.05 (1.03, 1.06)** |  |
|  |  | 2 | 71105 | 25618 | 425,943.1 | 60.1 | **1.11 (1.09, 1.13)** |  |
|  |  | 3 | 21782 | 8033 | 125,646.2 | 63.9 | **1.14 (1.12, 1.17)** |  |
|  |  | 4 | 3078 | 1164 | 16,946.5 | 68.7 | **1.20 (1.13, 1.27)** |  |
| **Sex** | **Male** | 0 | 277533 | 43533 | 2,028,059.9 | 21.5 | 1 (Ref.) | **<.0001** |
|  |  | 1 | 297307 | 51524 | 2,136,230.8 | 24.1 | **1.09 (1.07, 1.10)** |  |
|  |  | 2 | 144858 | 27945 | 1,014,327.4 | 27.6 | **1.20 (1.18, 1.22)** |  |
|  |  | 3 | 38102 | 8047 | 258,560.2 | 31.1 | **1.30 (1.27, 1.33)** |  |
|  |  | 4 | 4738 | 1106 | 30,567.0 | 36.2 | **1.43 (1.34, 1.51)** |  |
|  | **Female** | 0 | 102764 | 29072 | 693,562.7 | 41.9 | 1 (Ref.) |  |
|  |  | 1 | 141562 | 42185 | 939,946.7 | 44.9 | **1.04 (1.02, 1.05)** |  |
|  |  | 2 | 83989 | 26601 | 548,134.1 | 48.5 | **1.08 (1.06, 1.10)** |  |
|  |  | 3 | 25406 | 8356 | 162,442.3 | 51.4 | **1.12 (1.09, 1.15)** |  |
|  |  | 4 | 3372 | 1108 | 21,233.8 | 52.2 | **1.10 (1.04, 1.17)** |  |
| **BMI*** | **<25** kg/m^2^ | 0 | 187531 | 36704 | 1,337,886.7 | 27.4 | 1 (Ref.) | **<.0001** |
|  |  | 1 | 227466 | 50244 | 1,577,895.1 | 31.8 | **1.09 (1.07, 1.10)** |  |
|  |  | 2 | 123533 | 30500 | 828,690.2 | 36.8 | **1.16 (1.15, 1.18)** |  |
|  |  | 3 | 35711 | 9633 | 230,191.8 | 41.8 | **1.24 (1.21, 1.27)** |  |
|  |  | 4 | 4814 | 1335 | 29,891.4 | 44.7 | **1.24 (1.18, 1.33)** |  |
|  | **≥25** kg/m^2^ | 0 | 192766 | 35901 | 1,383,735.9 | 25.9 | 1 (Ref.) |  |
|  |  | 1 | 211403 | 43465 | 1,498,282.4 | 29.0 | **1.04 (1.03, 1.06)** |  |
|  |  | 2 | 105314 | 24046 | 733,771.2 | 32.8 | **1.12 (1.10, 1.14)** |  |
|  |  | 3 | 27797 | 6770 | 190,811.0 | 35.5 | **1.16 (1.13, 1.20)** |  |
|  |  | 4 | 3296 | 879 | 21,909.4 | 40.1 | **1.26 (1.18, 1.35)** |  |
| **Insulin** | **No** | 0 | 362901 | 67275 | 2614270.9 | 25.7 | 1 (Ref.) | 0.2475 |
|  |  | 1 | 410321 | 84472 | 2903765.4 | 29.1 | 1.06 (1.05, 1.07) |  |
|  |  | 2 | 207555 | 47332 | 1437936.5 | 32.9 | 1.13 (1.12, 1.15) |  |
|  |  | 3 | 55122 | 13448 | 373687.6 | 36.0 | 1.17 (1.15, 1.20) |  |
|  |  | 4 | 6568 | 1661 | 43327.9 | 38.3 | 1.19 (1.13, 1.25) |  |
|  | **Yes** | 0 | 17396 | 5330 | 107351.7 | 49.7 | 1 (Ref.) |  |
|  |  | 1 | 28548 | 9237 | 172412.1 | 53.6 | 1.03 (1.00, 1.07) |  |
|  |  | 2 | 21292 | 7214 | 124525.0 | 57.9 | 1.09 (1.05, 1.13) |  |
|  |  | 3 | 8386 | 2955 | 47315.0 | 62.5 | 1.17 (1.12, 1.22) |  |
|  |  | 4 | 1542 | 553 | 8472.9 | 65.3 | 1.19 (1.09, 1.30) |  |
| **DM Duration** | **<5 years** | 0 | 281332 | 47435 | 2,032,377.1 | 23.3 | 1 (Ref.) | **0.0002** |
|  |  | 1 | 315130 | 59126 | 2,239,147.4 | 26.4 | **1.07 (1.06, 1.08)** |  |
|  |  | 2 | 159389 | 32992 | 1,111,531.7 | 29.7 | **1.13 (1.12, 1.15)** |  |
|  |  | 3 | 42424 | 9399 | 289,944.1 | 32.4 | **1.18 (1.16, 1.21)** |  |
|  |  | 4 | 5146 | 1169 | 34,560.2 | 33.8 | **1.18 (1.11, 1.25)** |  |
|  | **≥5 years** | 0 | 98965 | 25170 | 689,245.4 | 36.5 | 1 (Ref.) |  |
|  |  | 1 | 123739 | 34583 | 837,030.1 | 41.3 | **1.06 (1.05, 1.08)** |  |
|  |  | 2 | 69458 | 21554 | 450,929.7 | 47.8 | **1.16 (1.14, 1.18)** |  |
|  |  | 3 | 21084 | 7004 | 131,058.5 | 53.4 | **1.24 (1.21, 1.28)** |  |
|  |  | 4 | 2964 | 1045 | 17,240.6 | 60.6 | **1.34 (1.26, 1.42)** |  |
| **CKD** | **No** | 0 | 348332 | 64575 | 2,498,864.5 | 25.8 | 1 (Ref.) | **0.0186** |
|  |  | 1 | 396470 | 81480 | 2,796,646.7 | 29.1 | **1.06 (1.05, 1.07)** |  |
|  |  | 2 | 202216 | 46110 | 1,394,940.9 | 33.2 | **1.14 (1.13, 1.16)** |  |
|  |  | 3 | 54469 | 13288 | 367,213.5 | 36.3 | **1.20 (1.17, 1.22)** |  |
|  |  | 4 | 6632 | 1699 | 43,509.7 | 39.1 | **1.23 (1.18, 1.29)** |  |
|  | **Yes** | 0 | 31965 | 8030 | 222,758.1 | 36.1 | 1 (Ref.) |  |
|  |  | 1 | 42399 | 12229 | 279,530.9 | 43.8 | **1.09 (1.06, 1.12)** |  |
|  |  | 2 | 26631 | 8436 | 167,520.5 | 50.4 | **1.17 (1.13, 1.20)** |  |
|  |  | 3 | 9039 | 3115 | 53,789.1 | 57.9 | **1.29 (1.23, 1.34)** |  |
|  |  | 4 | 1478 | 515 | 8,291.1 | 62.1 | **1.34 (1.23, 1.47)** |  |
| **OHA3** | **<3** | 0 | 340173 | 62065 | 2,442,990.5 | 25.4 | 1 (Ref.) | 0.0774 |
|  |  | 1 | 382425 | 77871 | 2,693,105.2 | 28.9 | 1.07 (1.06, 1.08) |  |
|  |  | 2 | 193340 | 43706 | 1,329,946.8 | 32.9 | 1.14 (1.13, 1.16) |  |
|  |  | 3 | 51826 | 12651 | 346,488.0 | 36.5 | 1.21 (1.18, 1.23) |  |
|  |  | 4 | 6408 | 1663 | 41,253.0 | 40.3 | 1.26 (1.20, 1.32) |  |
|  | **≥3** | 0 | 40124 | 10540 | 278,632.1 | 37.8 | 1 (Ref.) |  |
|  |  | 1 | 56444 | 15838 | 383,072.3 | 41.3 | 1.03 (1.01, 1.06) |  |
|  |  | 2 | 35507 | 10840 | 232,514.6 | 46.6 | 1.11 (1.08, 1.14) |  |
|  |  | 3 | 11682 | 3752 | 74,514.5 | 50.4 | 1.16 (1.12, 1.21) |  |
|  |  | 4 | 1702 | 551 | 10,547.8 | 52.2 | 1.17 (1.08, 1.28) |  |

*BMI ≥ 25 kg/m^2^was used to define obesity, following World Health Organization (WHO) recommendations for Asians.(*World Health Organization. (2000). The Asia-Pacific perspective: redefining obesity and its treatment.*)
